# Persistence in Phytopathogenic Bacteria: Do We Know Enough?

**DOI:** 10.3389/fmicb.2018.01099

**Published:** 2018-05-25

**Authors:** Paula M. M. Martins, Marcus V. Merfa, Marco A. Takita, Alessandra A. De Souza

**Affiliations:** ^1^Laboratório de Biotecnologia, Centro de Citricultura, Instituto Agronômico de Campinas, Cordeiropolis, Brazil; ^2^Department of Entomology and Plant Pathology, Auburn University, Auburn, AL, United States

**Keywords:** VBNC, phytopathogen, toxin-antitoxin systems, oxidative stress, crop diseases, persisters

## Abstract

Phytopathogenic bacteria affect a wide range of crops worldwide and have a negative impact in agriculture due to their associated economic losses and environmental impacts. Together with other biotic and abiotic stress factors, they pose a threat to global food production. Therefore, understanding bacterial survival strategies is an essential step toward the development of new strategies to control plant diseases. One mechanism used by bacteria to survive under stress conditions is the formation of persister cells. Persisters are a small fraction of phenotypic variants within an isogenic population that exhibits multidrug tolerance without undergoing genetic changes. They are dormant cells that survive treatment with antimicrobials by inactivating the metabolic functions that are disrupted by these compounds. They are thus responsible for the recalcitrance of many human diseases, and in the same way, they are thought to contribute to the survival of bacterial phytopathogens under a range of stresses they face in the environment. It is believed that persister cells of bacterial phytopathogens may lead to the reoccurrence of disease by recovering growth and recolonizing the host plant after the end of stress. However, compared to human pathogens, little is known about persister cells in phytopathogens, especially about their genetic regulation. In this review, we describe the overall knowledge on persister cells and their regulation in bacterial phytopathogens, focusing on their ability to survive stress conditions, to recover from dormancy and to maintain virulence.

## Introduction

Bacteria are able to cause diseases in a wide range of plants throughout the entire world (Strange and Scott, 2005; Kannan et al., 2015). These organisms, known as phytopathogenic bacteria, affect all food-producing plants, colonizing either their surface or tissues (Kannan et al., 2015). They cause symptoms such as spots, blights, cankers, tissue rots, and/or hormone imbalances that lead to plant overgrowth, stunting, root branching, and leaf epinasty, among others (Strange and Scott, 2005; Kannan et al., 2015). These issues impact plants on a qualitative and quantitative level, negatively affecting global food supplies (Kannan et al., 2015). Bacterial diseases of plants cause devastating damage to crops and significant economic losses. Collectively, they cause losses of over $1 billion dollars worldwide every year to the food production chain (Mansfield et al., 2012; Kannan et al., 2015). Together with other phytopathogens, such as fungi and viruses, and abiotic stress factors, including environmental degradation, climate change and chemical pollution, bacterial phytopathogens pose a global threat to agricultural food production. Thus, the development and employment of management approaches to overcome and suppress phytopathogenic bacteria, which includes mitigating their survival strategies, is imperative to global food security (Strange and Scott, 2005; Sundström et al., 2014). Therefore, studying and understanding the bacterial survival strategies is an essential step to find new possibilities to control plant diseases.

Bacteria have a variety of strategies to survive and thrive in the environment and in a host plant. Some of these strategies include the development of resistance against antimicrobials, the use of efflux pumps to detoxify the bacterial cell (Levy and Marshall, 2004; Wax et al., 2007), sporulation by gram-positive bacteria (Bobek et al., 2017), the employment of effector proteins to alter the host plant physiology and suppress its defense mechanisms (Jones and Dangl, 2006; Khan et al., 2017), the development of biofilms (Lewis, 2001; Fux et al., 2005; Danhorn and Fuqua, 2007), and the formation of persister cells (Lewis, 2007, 2008).

Persister cells are a small fraction of phenotypic variants within an isogenic population that exhibit multidrug tolerance without undergoing genetic changes (Keren et al., 2004b; Lewis, 2007, 2010; Maisonneuve and Gerdes, 2014). Approximately 0.001–0.1% of cells in a given bacterial population display the tolerance phenotype, and this number can increase up to 1% in stationary phase or in biofilms (Keren et al., 2004b; Lewis, 2008, 2010). Persister cells were first described by Bigger in 1944, who found that penicillin could not kill non-growing phenotypic variant cells of *Staphylococcus pyogenes* (*aureus*). Currently, it is known that the tolerance feature of persisters is related to a transient growth inhibition that results in the inactivation of the main cell functions (Lewis, 2010). Thus, persister cells are in a dormant state in which they are metabolically inactive; hence, they neither grow nor die in the presence of antimicrobial agents (Shah et al., 2006; Wood et al., 2013; Kim et al., 2017). Therefore, in contrast to resistance mechanisms, which act by blocking the interaction of an antimicrobial agent with its target, the tolerance of persister cells functions by preventing the damage of a cellular target by the bactericidal agent by shutting down the antimicrobial target in the cell. This occurs because persister cells have a low metabolic level and most of their metabolic functions that are disrupted by antibiotics are inactive (Shah et al., 2006; Lewis, 2007; Kim et al., 2017). Thus, bacterial populations developed two remarkably complementary and redundant strategies to avoid the action of antimicrobials: the employment of specific mechanisms of resistance and, when these fail, the formation of persister cells (Shah et al., 2006). While part of the population activates genes to directly respond to the stress, a smaller fraction is converted to a dormant state in a bet-hedging strategy to stress survival (Dörr et al., 2010).

From an evolutionary point of view, persistence is believed to have evolved as an alternative scenario in which metabolic balance is disturbed but the cell is kept alive. The growth inhibition in nutrient-deprived environments could be the key for bacterial survival before metabolic flux is so compromised that the restoration of growth is no longer possible (Radzikowski et al., 2017).

Through the cost of non-proliferation, persisters are able to guarantee the survival of the population, and so they are altruistic cells that forfeit growth for the benefit of the kin (Lewis, 2007). However, persister cells are not simply cells that do not grow. Fluoroquinolones, for instance, are able to kill non-proliferating cells, and the treatment of dormant cells with these compounds reveals the presence of persisters when the population is at stationary phase. This shows the difference between cells that are not growing and persister cells (Lewis, 2008). Persisters actually rely on genetic mechanisms to reach the dormant state and avoid stressful conditions; at the same time, they need a complementary mechanism to ensure the resumption of growth when conditions become favorable again (Lewis, 2008; Wood et al., 2013). When the antibiotic pressure ends, persister cells resume growth and originate a new population that has the same genotype as the original one. Therefore, the sensitivity of the population to antibiotics remains (Keren et al., 2004a). Regarding their development, persisters can be formed either stochastically, by fluctuations in gene expression, or actively, by responding to specific environmental cues (Balaban et al., 2004; Keren et al., 2004a; Dörr et al., 2009; Vega et al., 2012; Maisonneuve et al., 2013). Some of the factors that have been shown to induce persister cell formation include oxidative stress, starvation, DNA damage, macrophages, antibiotics, pH, and copper stress (Dörr et al., 2009, 2010; Hong et al., 2012; Muranaka et al., 2012; Wu et al., 2012; Bernier et al., 2013; Helaine et al., 2014; Ayrapetyan et al., 2015b).

The development of persister cells in bacteria is responsible for the recalcitrance of many chronic infections to antibiotics (Lewis, 2010). In certain human diseases, bacterial pathogens are able to survive even against high doses of antibiotics, even if they lack any mechanism of resistance against the applied antimicrobials (Harms et al., 2016). Persister cells can survive in biofilms or other protective niches and are thus the reason of many treatment failures (Levin and Rozen, 2006; Blango and Mulvey, 2010; Fauvart et al., 2011; Balaban et al., 2013; Bjarnsholt, 2013; Lebeaux et al., 2014). In the same way, it has been suggested that persister cells of phytopathogenic bacteria may recover and recolonize the environment after the action of an antimicrobial, leading to the development of disease all over again (Rodrigues et al., 2008; Muranaka et al., 2012).

Ever since the first report of viable but non-culturable cells (VBNC) in *Escherichia coli* and *Vibrio cholerae* (Xu et al., 1982), mostly as a consequence of the pioneering technique used to assess live/dead microbiota in seawater (Kogure et al., 1979), studies on persisters have increased continuously. At the same time, a prolonged debate has also endured on whether these cells are a survival strategy or a pre-death state (McDougald et al., 1998). This controversy is reflected by the nomenclature associated with persister cells, which still remains confusing, with a “dormancy continuum” state proposed (Ayrapetyan et al., 2015b) to encompass the VBNC (Xu et al., 1982) and persistence states (Hobby et al., 1942; Bigger, 1944), although other names for similar physiological states have also been proposed, such as active but non-culturable cells (Kell et al., 1998) and conditionally viable environmental cells (Lobitz et al., 2000). Finally, Kim et al. (2017) suggest that there are no substantial differences between VBNC and persister cells. The authors analyzed conditions known to induce both of these phenotypes in *E. coli* and concluded that between them, there are no evident differences concerning antibiotic tolerance, resuscitation (recovery) rates, morphology or metabolic activity. Another interesting finding is that some cells that stain as viable in light microscopy (due to their membrane integrity) are in fact, dead, since they have no internal cellular contents. It is not known how the internal contents of these cells leak, but it may have influenced the idea that many viable cells are not able to recover, when in fact, they are dead (Kim et al., 2017). There is still considerable debate on whether persisters are fully virulent, truly alive, dormant or dying. Indeed, studies (Kim and Wood, 2016) show that there are many misconceptions regarding persister cells in the literature.

In this review our aim was to focus on the current knowledge on this subject but specifically on the experimental data for phytopathogens. Their ability to survive under stress conditions by entering the persister state, the recovery conditions and the virulence maintenance were thoroughly reviewed in order to summarize the current knowledge about persister cell formation by phytopathogenic bacteria. We also analyzed the main factors that lead to their maintenance in the field even after chemical treatments or adverse environmental conditions.

## Viable but Non-Culturable and Persister Cell Occurrence in Phytopathogens

It is commonly accepted that bacteria present two dormant phenotypes, the VBNC and the persister state (Ayrapetyan et al., 2015b); the persister cells recover after the stress condition, whereas the VBNC cell population is reported not to recover (Ayrapetyan et al., 2015b; Kim et al., 2017). For phytopathogens, the majority of the works use the term “VBNC” to refer to the dormant phenotype; however, in all of these works, the authors were able to cultivate the cells after stress, or the cells were able to live in the host plant, indicating that these cells were in fact in the persister state. Actually, as mentioned above, there is no difference between VBNC and persister cells, where they both seem to be the same dormant phenotype but are considered as divergent because of an inaccurate evaluation of the viable cells (Kim et al., 2017). Therefore, even though many authors who work with phytopathogenic bacteria used the “VBNC” nomenclature, in the present review, we adopted only the term “persister” for reasons of clarity and conciseness.

The persister state in phytopathogens is still an understudied field when compared to that of human-associated bacterial pathogens, especially concerning its genetic regulation. The study of persisters offers a great potential for developing innovative ways for phytopathogen control as well as for uncovering specific stress conditions that induce cells to engage into persistence, which can be helpful in disease management planning. Thus, in the following sections, we will provide a summary of the state-of-the-art knowledge on persister cell induction among some bacterial phytopathogens, with the hope that it will enlighten future paths in phytopathology.

### Erwinia amylovora

The causative agent of fire blight, *Erwinia amylovora* (syn. *Micrococcus amylovorus*, *Bacillus amylovorus*) is a ɣ-proteobacterial pathogen not only of economic impact but also of historical relevance. It was the first bacterium to be proven as the cause of a plant disease and is therefore considered the first phytopathogen described. It is easily disseminated by insects, especially those that visit flowers but can also be spread by wind that carries the bacterial ooze. It infects *Rosaceae* plants, including ornamentals and bushes, but its major impact is on fruit production. Global numbers on its economic impact are unknown, but epidemics are frequent in pear and apple fields (Thomson, 1986; Mansfield et al., 2012; Santander et al., 2014a; CABI, 2017a).

It is a non-obligate pathogen that is able to live within a wide temperature range (4–37°C), and its epiphytic growth is restricted to the flower stigma (Thomson, 1986). *E. amylovora* experiences periods of stress, such as starvation, throughout its life cycle, and its physiological responses seem to be linked to the temperature of the surrounding environment (Santander et al., 2014b). Starving cells (122 days old) of *E. amylovora* enter into persistence at 28 and 4°C but prefer to maintain their culturability at 14°C. This cyclic behavior seems to be common for other pathogens and may be responsible for the periodic cycles of disease throughout the year (Santander and Biosca, 2017).

The seasonality of *E. amylovora* also relies on its ability to survive in infected tissues, such as stem cankers, where it waits for better environmental conditions to multiply and spread. It is accepted that during its overwintering, *E. amylovora* faces a nutritional shortage, and starvation stress responses may be triggered to enhance its chances to survive. One of the major regulators for “famine” in bacteria is the RpoS sigma factor, which is involved in many other stress responses and is widely present throughout the prokaryotes. In *E. amylovora*, *rpoS* deletion mutants (*rpoS^-^)* entered into the persister state faster than wild-type cells. They were unable to trigger the normal starvation stress mechanism, leading to a more pronounced decrease in the number of viable and culturable cells (Santander et al., 2014a).

Starvation stress in *E. amylovora* is also connected to oxidative stress responses. *E. amylovora* has two catalase genes, *katA* and *katG*, which when deleted (*katAG-*) lead the cells to enter into the persister state faster, although the growth of this mutant in solid culture media remains normal. Additionally, when these catalases are overexpressed or added to the plating medium, the number of growing cells is higher, demonstrating that catalase contributes to the maintenance of the culturability of *E. amylovora* under these conditions (Santander et al., 2017). The relationship between catalase activity and culturability is not new, however. For some bacteria, such as *Vibrio vulnificus* (Kong et al., 2004) and the phytopathogen *R. solanacearum* (Kong et al., 2014), this enzyme can help reactivate persister cells (as will be discussed further), thus starvation stress is tightly linked to the increase of internal oxidative stress (McDougald et al., 2002). Moreover, catalase and peroxidase genes are usually controlled by RpoS (Santander et al., 2017), highlighting the connection between starvation and oxidative stresses.

The chemical control of fire blight relies basically on antibiotics and copper applications. The latter is used for fire blight control mainly in Europe, since in the United States, antibiotics are still allowed to be sprayed in orchards, a practice that is highly controversial due to resistance development, not to mention the destruction of many other non-target bacteria, and the concerns that resistance genes could be transmitted to animal and human pathogens. Therefore, caution is required in such practices in agriculture (McManus et al., 2002; McGhee and Sundin, 2011).

Cupric compounds induced persister cell formation in *E. amylovora* (Ordax et al., 2006) in a concentration-dependent manner. Cells invariably entered the persister state but reached unculturable levels at time zero when 0.05 mM of copper was added, although viable cell counts by live/dead assay showed a high proportion of living bacteria at 270 days. Recovery of persister cells was accomplished after the addition of chelating agents such as EDTA, and no difference in pathogenicity could be detected between control and reactivated cells. When mature apple calyces were inoculated, copper applications induced the unculturable state at even lower concentrations (0.1 and 0.01 mM). Chlorine, a bactericide used to disinfect tools (CABI, 2017a), also induced persistence in *E. amylovora* (Santander et al., 2012), a phenomenon that has already been demonstrated for other human-associated bacteria (Oliver et al., 2005).

It seems, however, that the best reactivation factor for *E. amylovora* persister cells is the host plant (Santander et al., 2012). Regardless of the stress applied (chlorine and starvation), inoculation in pear plantlets always recovered *E. amylovora* cells, but the chlorine-stressed cell recovery was astonishing, since after just 5 min in contact with 0.7 ppm of chlorine, culturability reached undetectable levels in solid media, but viable cell counts showed that approximately 10^6^ cells mL^-1^ were stably present for at least 24 h. In addition, when inoculated in its host, these viable cells could colonize and induce disease symptoms normally, indicating that the cells recovered. This demonstrates that the control of diseases in the field is much more complex than what is observed *in vitro*.

Overall, persistence studies in *E. amylovora* not only show the occurrence of the unculturability phenomena under different stress situations, but also highlight the potential risks associated with the under-detection of real viable cells, especially when dealing with chemical control and the spread of the phytopathogen through fruit transportation (Ordax et al., 2009; Santander et al., 2012).

### Ralstonia solanacearum

*Ralstonia solanacearum* (syn. *Pseudomonas solanacearum, Burkholderia solanacearum*) (Yabuuchi et al., 1995) is a phytopathogenic β-proteobacterium that infects many different plants, causing bacterial wilt and leading to enormous economic losses in commercial production. It is one of the most destructive pathogens to commercial crops (Mansfield et al., 2012), causing extensive damage in tobacco, potato, tomato and banana crops; it is a pathogen for which there are no chemical controls available, and many measures are required to prevent its spread (Yuliar et al., 2015).

*R. solanacearum* is a soil-borne bacterium that attacks plants by the roots, eventually causing death after spreading through the vascular system. The bacteria are then released again into the soil, where they are assumed to live until they reach the next host. Soil oligotrophy requires special skills for microorganisms that thrive in this environment, at least until they again find the most welcoming rhizosphere (Grey and Steck, 2001). Similar lifestyles for other phytopathogens point to the same strategy, that is, an encounter between the bacteria and its host provides the best scenario for a growth recovery (Santander et al., 2012).

Grey and Steck (2001) showed indeed that this pathogen enters the persister state in sterile soil, while retaining its virulent potential. The authors showed that in sterile soil, an initial inoculum (10^11^ cells kg^-1^ soil) is undetected by culturing after 3 days, and in copper-supplemented soil, the culturability threshold is less than 2 days. However, the live-dead assays show that the cell count decreases by only 1 log, to 10^10^ cells kg^-1^ soil, and is kept stable for at least 30 days, which indicates that culturable cell counts are misleading as an assessment for the presence of this phytopathogen since plating counts do not represent the viable bacterial population. Additionally, when germinating tomato seeds were added to soil where no culturable cells could be detected, the plants exhibited wilt symptoms after 15 days, indicating that persister cells could be reactivated and retain their virulence after exiting the persister state. In this case, the rhizosphere seems to be the stimulus required by the cells to restart their growth, and culturable cells were detected even at 1 cm away from the roots, although the chemical signals are still unknown. This was the first report showing that a phytopathogen can be reactivated after stress.

Despite the initial work by Grey and Steck (2001), the long-term starvation stress responses of *R. solanacearum* were only studied years later, in water microcosms (200 mL of sterilized water from rivers) (Alvarez et al., 2009). During the first 6 months, the initial bacterial inoculum was of 10^7^ cells mL^-1^ in live/dead assays, with similar numbers of culturable cells (10^6^ CFU mL^-1^). However, from the first to the 4th year, the culturable cells count dropped to 10^4^ and 10^3^ CFU mL^-1^, even though live/dead tests showed that 10^7^ cells mL^-1^ still presented an intact membrane. The starved cells progressively shifted from a normal bacilli structure to a coccoid cell shape and showed a more aggregative behavior, probably to improve their ability to acquire nutrients and to protect themselves from predation. These results suggest that under prolonged starvation, a substantial proportion of the *R. solanacearum* population enters the persister state as a survival strategy in order to survive under harsh environmental conditions before reaching more favorable situation to activate their growth. After the 4-year starvation experiment, *R. solanacearum* remained virulent and infective, since tomato plants developed wilt symptoms when they were stem-inoculated or irrigated with the water microcosms.

Low temperatures also constitute a type of stress that induces persister formation (van Elsas et al., 2000). There is evidence that *R. solanacearum* cells can enter in an unculturable state in water bodies during winter, which is of special interest in temperate countries. A seasonal oscillation of *R. solanacearum* in water flows, consistent with the entry of the fully active cells in summer into a persister state during winter, was reported in the Netherlands (van Elsas et al., 2001) and Spain (Caruso et al., 2005). This may be the reason why this pathogen remains undetected during the coldest months of the year but is still able to induce symptoms in tomato plants when contaminated water is used in irrigation (Caruso et al., 2005).

Curiously, *R. solanacearum* subjected to cold stress could be reactivated by the addition of catalase (Kong et al., 2014), similar to what is observed for *E. amylovora* (Santander et al., 2017). Temperature-stressed non-culturable cells of *R. solanacearum* were supplemented with 1,000 UmL^-1^ of catalase and kept at 30°C for up to 3 days, when colonies could be detected in solid media. Converging results were found for copper-stressed persister *R. solanacearum* (Um et al., 2013) cells that showed elevated levels of H_2_O_2_ in comparison to those that were culturable. The addition of other peroxide-degrading compounds, such as sodium pyruvate resulted in a similar outcome, increasing the number of culturable *R. solanacearum* cells (Imazaki and Nakaho, 2009) These results are in agreement with what is known for other microorganisms, where the ability to detoxify cells under oxidative stress may be a pivotal mechanism and one of the most important factors that reactivate the ability to grow (Mizunoe et al., 1999; Kong et al., 2004).

Among the most important triggers of persister cells are toxic compounds, such as pesticides. Cupric compounds are widely used in agriculture as bactericides and are common stressor agents faced by phytopathogens. Copper kills the majority of viable *R. solanacearum* cells, but the remaining cells enter a persister state in a concentration-dependent manner. Therefore, 5 μM copper is sufficient to induce persister cell formation of up to 99.9% of the remaining viable cells in the course of 2 weeks, reaching 100% when 500 μM copper is used (Grey and Steck, 2001). Similar results were obtained by Um et al. (2013) after the addition of high concentrations of copper. The authors also observed a correlation between copper stress and aggregative behavior as well as increased concentrations of H_2_O_2_, which is believed to be the trigger for the suppression of colony formation in solid growth media (Um et al., 2013). This is also consistent with persister cell formation under other stress conditions (Alvarez et al., 2009).

Um et al. (2013) also verified that *R. solanacearum* responds to copper stress by increasing the total amount of DNA per cell and decreasing the RNA content to undetectable levels within just 24 h after copper addition. On the other hand, control cells kept a regular amount of RNA throughout the experiment. Transcriptome analysis revealed the down-regulation of catalase and peroxidase genes together with up-regulation of the Dps protein, which is involved in DNA protection under stressful conditions. It is important to mention that residual amounts of copper in field conditions are higher than those used by Um et al. (2013), suggesting that persister cells could naturally occur in the environment.

One of the most interesting findings for persister cells in *R. solanacearum* is that they are formed while the bacteria are still inside the host, as the disease progresses and nutrient availability decreases. *R. solanacearum* enters the persister state in varying degrees during infection, but eventually it reaches 99% when extensive necrosis occurs (Grey and Steck, 2001), suggesting that even before the phytopathogen reaches the soil, it is already prepared to survive the adverse conditions it is about to face.

Overall, the current knowledge of persister cells of *R. solanacearum*, although controversial in some points, is well established. It is important to highlight that this pathogen is not native to European soils, and as such, cold stress may be one of the few soil conditions that this pathogen has not been evolutionarily selected for. Overwinter ability is a special requirement for lineages to thrive in open areas, especially in temperate regions, and since *R. solanacearum* wilt is still a major problem to many crops worldwide, it is reasonable to conclude that the induction of persistence is a way for this phytobacterium to survive stress.

### Xylella fastidiosa

*X. fastidiosa* is a ɣ-proteobacterium that lives only in the xylem of infected plants and in the foregut of sharpshooter insect vectors, which transmit it directly to the xylem of host plants (Almeida et al., 2014). This bacterium is associated with many plant diseases that impact economically important crops worldwide, including citrus, grapevine, plum, almond, peach, coffee, blueberry and more recently, olives (Hopkins and Purcell, 2002; Saponari et al., 2013). Additionally, *X. fastidiosa* colonizes many grasses and weeds without causing disease, which serve as a source for bacterial spread (Hopkins, 1989). The main symptoms caused by *X. fastidiosa* in diseased plants are leaf chlorosis, marginal scorching and/or dwarfing, depending on the host (Hopkins and Purcell, 2002). Its main mechanism of pathogenicity is considered to be the systemic colonization of infected plants’ vessels by multiplication and movement of the bacterium, followed by biofilm formation, which blocks the xylem vessels and impairs the movement of water and nutrients within plants (Chatterjee et al., 2008). Bacterial growth in the biofilm state is also required for the insect vector to acquire the bacterium from infected plants (Chatterjee et al., 2008). As mentioned above, cells in a biofilm have adaptive advantages in the environment, such as increased resistance to a wide range of antimicrobial compounds and the induction of persister cell formation (Mah and O’Toole, 2001; Lewis, 2007; Rodrigues et al., 2008; Muranaka et al., 2012).

The first evidence of persister cell formation in *X. fastidiosa* was found in biofilm cells under copper stress (Rodrigues et al., 2008). It was observed that even after treating *X. fastidiosa* cells with an inhibitory concentration of copper, the cells still harbored good quality RNA, and genes could still be expressed, suggesting persister cell formation (Rodrigues et al., 2008). This result was later confirmed by Muranaka et al. (2012), who showed that *X. fastidiosa* forms persister cells when treated with inhibitory concentrations of both copper and tetracycline, with a survival rate of approximately 0.05% for cells grown in either condition. In addition, the pretreatment of the mature biofilm of *X. fastidiosa* with a subinhibitory concentration of copper prior to the treatment with the inhibitory concentration of this element, increased the formation of persisters by 26-fold. This ability was not found in similarly treated planktonic cells (Muranaka et al., 2012). Another study published by Navarrete and De La Fuente (2014) also showed that *X. fastidiosa* forms persister cells even without the presence of an antimicrobial compound. However, when cells were treated with a subinhibitory concentration of zinc, the process of persister cell formation was hastened (Navarrete and De La Fuente, 2014). These studies show the ability of *X. fastidiosa* to form persister cells in both regular growth conditions and under antimicrobial stress and highlight the threat that these cells represent for agriculture, since antimicrobial compounds fail to kill the whole population, and they can recover after the stress (Rodrigues et al., 2008; Muranaka et al., 2012).

*X. fastidiosa* is one of the few phytopathogens in which the molecular mechanisms of persister cell formation have been investigated. In addition to studying the formation of persister cells by *X. fastidiosa* exposed to inhibitory concentrations of antimicrobials, Muranaka et al. (2012) also analyzed the transcriptional profile of this bacterium when subjected to copper and tetracycline stress conditions. This led to the repression of genes related to metabolic functions and movement, and the induction of specific resistance genes against each antimicrobial. In addition, several toxin-antitoxin (TA) systems were induced when *X. fastidiosa* was treated with both antimicrobial compounds (Muranaka et al., 2012). TA systems consist of a pair of genes located in the same operon, in which one encodes a stable toxin that disrupts an essential cellular process leading to growth arrest, and the other encodes the cognate labile antitoxin that prevents the toxicity of the system (Wang and Wood, 2011; Gerdes and Maisonneuve, 2012). In general, the antitoxin is able to regulate the expression of its own operon by binding to a palindromic sequence in the promoter region, acting as a transcriptional repressor of the TA system (Wang and Wood, 2011). TA systems are highly expressed in persister cells, and they are primarily responsible for the formation of this phenotype because they induce the dormant state required for cells to become persisters, enabling them to escape the action of antimicrobials and other stresses (Keren et al., 2004b; Shah et al., 2006; Lewis, 2008; Wang and Wood, 2011). The activation of the system occurs through the action of proteases that are induced during stressful conditions; these degrade the labile antitoxin and release both the toxin and the promoter region of the system, allowing its transcription and expression, which will result in growth inhibition and persister cells formation (Christensen et al., 2004; Maisonneuve and Gerdes, 2014).

Muranaka et al. (2012) observed that when *X. fastidiosa* was treated with the inhibitory concentration of copper, besides forming persister cells, 12 out of 65 TA systems were induced, with *mqsRA* being the most induced under this condition. This TA system was initially described in *E. coli*, where it was shown to be highly associated to persister cells and biofilm formation (Wang and Wood, 2011). The MqsRA TA pair is composed of the MqsR toxin, which is a ribonuclease (Brown et al., 2009) that cleaves mRNA specifically at GCU sites (Yamaguchi et al., 2009) and requires the proteases Lon and ClpXP for its toxicity (Kim et al., 2010); and the MqsA antitoxin, which binds to MqsR by its N-terminal domain and to DNA via a helix-turn-helix (HTH) motif in its C-terminal domain (Brown et al., 2009). In *X. fastidiosa*, MqsR degrades mRNAs primarily by cleaving them at GCU sites, and MqsA inhibits the action of MqsR by directly binding to it (Lee et al., 2014; Merfa et al., 2016). Even though the binding of MqsA to its own promoter has not been assessed in *X. fastidiosa*, in this organism, the antitoxin has the same Asn97 and Arg101 residues that are used by the antitoxin from *E. coli* to bind to DNA in the palindromic sequences 5′-ACCT (N)3 AGGT and 5′-TAACCT (N)3 AGGTTA (Yamaguchi et al., 2009; Brown et al., 2011; Merfa et al., 2016). In addition, in the promoter region of *MqsRA* in *X. fastidiosa*, there is a 5′-TAACCT (N)3 AAGTTA sequence that is very similar to the palindromic sequence located in the promoter of *mqsRA* in *E. coli*. Thus, the regulation of *mqsRA* transcription by MqsA in *X. fastidiosa* probably occurs in a similar manner as in *E. coli* (Merfa et al., 2016), suggesting a conserved mechanism in plant and human bacterial pathogens.

The *mqsR* toxin is the most induced gene in persister cells of *E. coli*, and the MqsRA TA system was the first system in which its deletion resulted in decreased persister cell formation (Shah et al., 2006; Kim and Wood, 2010). Deleting MqsR alone also resulted in decreased persister cell formation, and as expected, the production of *MqsRA* increased persistence (Kim and Wood, 2010). Likewise, the overexpression of *mqsR* also increased persister cell formation in *X. fastidiosa* cells treated with subinhibitory and inhibitory concentrations of copper, thus confirming the role of this TA system in persister cell formation in this bacterium. The formation of persisters by *X. fastidiosa* overexpressing *mqsR* was observed both by cell survival rate analysis and by quantifying the proportion of elongated cells in the population treated with different concentrations of copper (Merfa et al., 2016). Elongated cells are a good indicator of persister cell formation; since persisters have decreased cell metabolism, cells do not divide in this state, leading to their elongation (Balaban et al., 2004; Maisonneuve et al., 2013). As discussed above, the expression of TA systems lead to the persister phenotype because they induce cell dormancy (Wang and Wood, 2011). For *MqsRA*, the decrease in cell metabolism is due to the mRNA cleavage by MqsR. Thus, *MqsRA* induces dormancy by decreasing translation (Wood et al., 2013).

Lastly, although persister cell formation is thought to be the main physiological function of TA systems, another intriguing role is in controlling other phenotypic features in the cell. MqsR, specifically, is a motility quorum sensing regulator directly related to biofilm formation in *E. coli*. This toxin is induced in biofilms, and its deletion decreases biofilm formation (Ren et al., 2004; González Barrios et al., 2006). The same was observed for *X. fastidiosa*, in which deletion of *mqsR* also reduces biofilm formation (Lee et al., 2014), while its overexpression increases biofilm formation and reduces cell movement, abolishing bacterial pathogenicity in citrus (Merfa et al., 2016). In *E. coli*, it is known that MqsRA controls biofilm formation and other features of the cell by differential mRNA decay caused by the toxin and by the unique property of MqsA to bind to the promoter region of other genes besides its own to control their expression (Wang et al., 2011; Wang and Wood, 2011; Soo and Wood, 2013). However, in *X. fastidiosa*, the molecular mechanism by which MqsRA controls sessile and motile growth is not fully understood. In addition to regulating persister cell formation, MqsRA may play a key role in the adaptation and survival of *X. fastidiosa*, since its lifestyle involves sessile and motile growth, and the formation of persister cells is important for colonization and survival within its hosts (Chatterjee et al., 2008; Muranaka et al., 2012; Merfa et al., 2016).

### *Xanthomonas* spp.

One of the most important genera among the phytopathogens, *Xanthomonas* species cause diseases in virtually all economically important crops: orange, cassava, tomato, pepper, crucifers, cotton, rice, beans and grapes are examples of host plants usually affected. They are rod-shaped ɣ-proteobacteria, distributed worldwide and are easily spread by rain, winds, contaminated plant material and agricultural tools (Graham et al., 2004). Control measures are restricted to copper applications in field, which still has limited results, despite the impacts to soil and plant toxicity (Ruyters et al., 2013; Hippler et al., 2018). Moreover, the use cupric compounds as stressor agents provided the first evidence of persister cells formation in *Xanthomonas* (Ghezzi and Steck, 1999; del Campo et al., 2009).

*Xanthomonas campestris* pv. *campestris* is the etiological agent of black rot in crucifers, a disease present worldwide (CABI, 2017b). *X. campestris* enters the persister state after just 2 days in a liquid microcosm containing salts and supplemented with copper sulfate. Concentrations as low as 0.005 mM of copper are enough to suppress colony formation in this bacterium, while viability is maintained at 10^6^ cells mL^-1^. Additionally, the liquid microcosm alone could induce persistency at day 39, which was much later than that of the copper-supplemented medium. Similar results were observed in copper-supplemented sterile soil, although to a lesser extent than on liquid microcosms. For 48 days, culturable cells could be recovered, but always at lower numbers than those of viable cells. Similar to what happens in liquid microcosms, the culturability of *X. campestris* in copper-free control soil decreases, but copper addition facilitates the formation of persisters, since culturability decreased one additional log (Ghezzi and Steck, 1999).

Another important bacterium is the citrus canker etiological agent, *Xanthomonas citri* subsp. *citri*, which, as many other phytopathogens, has no chemical control measures other than the application of cupric compounds. Evidence of persister cell formation was obtained when *X. citri* was exposed to 135 μM copper (an amount three times higher than the concentration applied in one field treatment) for 10 min, rendering the cells unculturable, but when subsequently infiltrated into a susceptible host, the results revealed that 1% of the population was still pathogenic (del Campo et al., 2009).

Copper-resistant strains of *Xanthomonas* are continuously appearing (Richard et al., 2017b; Gochez et al., 2018), but this should not be the only concern for field applications of cupric compounds. Although just two *Xanthomonas* strains to date have been shown to enter the persister cell state in response to copper induction, there are undoubtedly more *Xanthomonas* strains and more conditions that will be listed as potential inducers of persistence for the phytopathogens of this genus.

### Clavibacter michiganensis

The causative agent of the bacterial canker of tomato, *Clavibacter michiganensis* subsp. *michiganensis* is an actinobacterium that is transmitted by seeds and causes substantial economic losses worldwide. It is the first gram-positive phytopathogen studied under different stress conditions in order to assess the existence of persister cells (Jiang et al., 2016). Similar to the situation with *E. amylovora*, some countries permit the use of antibiotics to control the bacterial canker of tomato in the field, but cupric compounds are still widely used. *C. michiganensis* becomes unculturable 37 days after copper treatment using concentrations as low as 0.05 μM, but unculturability is achieved in only 2 h at 50 μM copper sulfate. Unculturable cells are able to recover and multiply but do not induce symptoms when inoculated into tomato plants (Jiang et al., 2016).

### Pseudomonas syringae

*Pseudomonas syringae* is a γ-proteobacterium present worldwide. It seems to be primarily an epiphytic, opportunistic pathogen that lives on healthy plant surfaces, even on non-host plants (Romantschuk et al., 1997).

In the early 1990s, the majority of reports on persister cells were devoted to aquatic environments (van Overbeek et al., 1990; Byrd et al., 1991), and the occurrence on terrestrial microbes was practically unknown. One of the first studies on the plant phylloplane was on *P. syringae*, which was observed to enter an unculturable state (75% of the bacteria) after 80 h on bean plants, probably due to starvation, even though viable cells could also be identified (Wilson and Lindow, 1992). Years passed until other persistence-inducers in *P. syringae* were studied, and surprisingly, one compound was identified as acetosyringone (Mock et al., 2015). This is a phenolic compound produced by plants that has (besides other functions) antioxidant activity under oxidative stress conditions (Shalaby and Horwitz, 2015). When it is oxidized by H_2_O_2_ and peroxidases, the resulting micro-environment has a higher redox potential, which is not favorable for bacterial growth. In the presence of 50 μM of acetosyringone (plus H_2_O_2_ in an oxidation reaction mix), the number of culturable cells of *P. syringae* pv. *syringae* was reduced to 0.01% (initial inoculum of 10^7^ CFU mL^-1^) after 2 h, with the unculturability effects lasting at least 7 h. This work reinforces the importance of plant-associated molecules – and not only environmental stress – as potential inducers of bacterial persistence (Mock et al., 2015).

Using similar conditions, a global gene expression study was performed on unculturable cells of *P. syringae* pv. *syringae* (Postnikova et al., 2015). After 3 h in contact with 100 μM of acetosyringone oxidation reaction mix, over 900 differentially expressed genes were found. The majority of these genes could be linked to oxidation stress responses. However, since 99% of the cells were unculturable at this point, it is highly probable that the other expressed genes are related to the interruption of cellular growth.

On the other hand, virulence-associated genes were significantly down-regulated. It appears that the entry into the unculturable state also drives the persister cells to halt the synthesis of pathogenesis-related proteins of the Type III secretion system, phytotoxin and transport. Among the up-regulated categories are many genes involved in metabolism and transport (carbohydrate, quaternary ammonium, polyamine), chemotaxis/chemosensing, peptidoglycan/cell wall polymers and energy generation (Postnikova et al., 2015).

In the energy generation category, the presence of one aconitase gene (AcnA) is of special interest. AcnA aconitase is preferably used over AcnB during the stationary phase and oxidative stress (Jordan et al., 1999). It is a bifunctional protein, participating of the Krebs cycle but also in iron regulation metabolism. When in the presence of superoxide molecules, the iron present into the *iron-sulfur cluster* (Fe-S) of the aconitase is lost (Imlay, 2006), rendering this protein inactive (Walden, 2002). In the absence of extra iron to reconstitute the Fe-S cluster, this protein blocks the translation of ferritin mRNA, which causes a decrease in iron storage and consequently enhances the availability of this metal. Postnikova et al. (2015) presented the hypothesis that during acetosyringone oxidation by H_2_O_2_, aconitase is induced to assume its function in the iron metabolism, setting aside the Krebs cycle. The authors conclude that iron deployment within the stressed cell and its downstream metabolism could be an important characteristic of these cells. Acetosyringone oxidation may require higher levels of AcnA in the bacterial cell, which explains why its gene is up-regulated.

Future work certainly will improve the knowledge on the gene regulation networks and the physiological states associated with unculturability and persister cell formation in phytopathogenic bacteria that hopefully will also enlighten the path for alternative control measures for diseases in agriculture.

## Genetic Mechanisms That Induce Persister Cells in Phytopathogenic Bacteria

In general, genetic regulation in persister cells is quite similar among all bacteria, regardless to their lifestyle, revealing that this is a conserved prokaryotic response to stress among non-sporulating bacteria (Ayrapetyan et al., 2015a). Still, the molecular mechanisms that underlie persister cell formation are still largely unknown (Pinto et al., 2015). Even though the studies on genetics and physiology of persisters are scarce for phytopathogens, the few works that aimed to understand the molecular mechanisms behind persister cell formation show underlying similarities.

Only two global gene expression studies have focused on the molecular mechanisms involved in the regulation of persistence in phytopathogens (Muranaka et al., 2012; Postnikova et al., 2015). *X. fastidiosa* is unique in that persister cell formation in this phytopathogen involves the type II TA system (Muranaka et al., 2012; Merfa et al., 2016). In *E. coli*, Lon protease activity is required for persister cell formation by the type II TA system (Lewis, 2010; Maisonneuve et al., 2011; Maisonneuve and Gerdes, 2014). The degradation of the antitoxin by Lon during stress is the trigger for the activation of the TA system and the response of the cell, culminating in growth arrest and the persister phenotype due to the action of the toxin (Christensen et al., 2004; Maisonneuve and Gerdes, 2014). However, it is not currently known if this molecular mechanism also regulates persister cell formation in *X. fastidiosa*, since Muranaka et al. (2012) did not find Lon or any other protease induced in persister cells of this bacterium. However, the role of Lon in regulating persister cell formation in *X. fastidiosa* cannot be ruled out because in their study, only one timepoint was evaluated, and although no mRNA was detected, the protein could still be present. The mechanism by which *X. fastidiosa* enters the persister state is not well understood, but gene expression profiles have demonstrated a general up-regulation of TA systems in persister cells. Indeed, one of these TA systems, *mqsRA*, is involved in the increased formation of persister cells under copper stress in *X. fastidiosa* (Merfa et al., 2016). The overexpression of MqsR also led to a higher cell survival and the formation of persister cells in *X. fastidiosa* under copper stress, which was associated with the increased occurrence of elongated cells (Merfa et al., 2016). The mechanism by which *mqsRA* induces the formation of persister cells has already been described in *E. coli* (Wang and Wood, 2011). According to Merfa et al. (2016), persistence in *X. fastidiosa* could play a role in survival under environmental stresses, allowing for recovery and consequent recolonization after the end of the stress condition.

In addition to *X. fastidiosa*, *P. syringae* is the only other phytopathogen in which a global gene expression study of persistence was done, using oxidized acetosyringone as a persistence inducer (Postnikova et al., 2015). This study tried to mimic an *in planta* defense response, which could induce persistence in this bacterium (Mock et al., 2015).

Although the participation of TA systems in this process was not mentioned, one of the main regulatory responses of *P. syringae* includes the induction of an aconitase A gene (*acnA*) (Postnikova et al., 2015). Under oxidative conditions, AcnA participates in iron regulation (Imlay, 2006) that may result in persistence (Postnikova et al., 2015). Curiously, in *Xanthomonas campestris* pv. *vesicatoria*, the aconitase B gene is involved in its growth in plants and symptom development (Kirchberg et al., 2012), and it is co-transcribed with a TA system, which seems to be conserved among *Xanthomonas* spp (Martins et al., 2016). These findings indicate that aconitase expression, TA systems and persister formation are connected, and further studies are needed to better understand the participation of these molecular mechanisms in phytopathogenic bacteria.

Reactive oxygen molecules are common among stress responses and the subsequent triggering of persistence in many bacteria (Kong et al., 2004; Imazaki and Nakaho, 2009). Reactive oxygen species (ROS) are highly detrimental to the multiplication of microbes and their survival. Many important enzymes are sensitive to superoxide ions, and oxidative environments can impair the acquisition of important elements, such as iron (Imlay, 2006). Not surprisingly, as a control measure for infections, plant and animal defense systems rely on ROS bursts as a primary strategy, which successful pathogens are expected to handle and survive. Common adverse conditions found in the field, such as copper and cold stress, induce internal oxidative stress that mounts downstream changes in cell physiology (Hippler et al., 2018). Catalase-deficient strains of *E. amylovora* become persistent faster than those strains that overexpress this enzyme or if it is added to the culture medium (Santander et al., 2017). Similar behavior was also observed in *R. solanacearum* (van Overbeek et al., 2004; Um et al., 2013; Kong et al., 2014) and many non-phytopathogens (Mizunoe et al., 1999; Chaiyanan et al., 2007). Glutathione S-transferase, a protein also involved in oxidative stress protection (Allocati et al., 2009), was found to be overexpressed in a *Vibrio vulnificus* mutant strain that did not enter into an unculturable state (Abe et al., 2007). The wild-type strain, however, retained its culturability when this enzyme was added to the culture medium, which suggests that other enzymes besides catalase are working on the culturability balance in a wide range of bacterial strains (Abe et al., 2007). Overall, the results suggest that the ability of cells to restore internal balance from oxidative stress is a central metabolic determinant of culturability.

**FIGURE 1:**
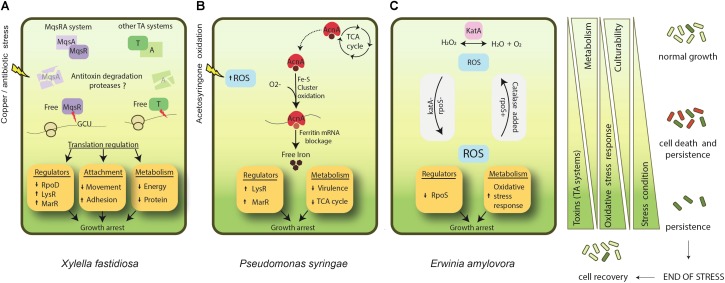
Known mechanisms of persister formation in phytopathogenic bacteria. **(A)** *X. fastidiosa*, under copper/antibiotic stress, presents induction of MqsRA and other TA systems. Degradation of antitoxins leads to an increase of free MqsR and other toxins in the cell. MqsR degrades mRNA specifically at GCU sites, and together with the action of other toxins, it regulates translation of proteins. In general, this process involves repression of RpoD, and induction of other regulators such as LysR and MarR. In addition, metabolism and cell movement are repressed, and adhesion is increased. Together, they lead to cell growth arrest and ultimately to persister cell formation. **(B)** In *P. syringae* acetosyringone oxidation leads to an increase in ROS formation. In parallel, aconitase (*acnA*) involved in the tricarboxylic acid (TCA) cycle is up-regulated. AcnA is a bifunctional protein that, under an oxidative environment, switches its function from the TCA cycle to act as a regulator of ferritin after the oxidation of its Fe-S cluster. This ultimately impacts iron metabolism, increasing free iron. Overall, this process involves induction of LysR and MarR regulators and repression of virulence factors and the TCA cycle, triggering cell growth arrest and persistence. **(C)** *E. amylovora* studies on persister cells are based on *katA* (catalase) and *rpoS* (sigma factor 38) mutant phenotypes. Oxidative stress is supposed to be one of the main triggers of persistence for this bacterium. *katA*- and *rpoS*- strains present higher level of ROS and enter faster into unculturability. The addition of catalase (either by super expression or supplemented in growth media) decreases ROS level and delays the entrance into persistence. ↑ indicates induction and ↓ indicates repression. Light green indicates condition of normal growth and dark green represents a state of persistence induction. Cells follow the same representation of green shades. The red color indicates dead cells. After end of stress the population recovers and enters into normal growth state.

In any case, persisters are formed through different mechanisms and further investigation is required to unveil the entire molecular mechanism behind persister cell formation in phytopathogenic bacteria (**Figure 1**).

## The “Super-Phytopathogenic” Bacteria and Implications of Persistence in Agriculture

Currently, there is a major concern regarding ‘superbugs’, which are bacteria resistant to commonly used antibiotics, representing a serious global threat to human health (Vargiu et al., 2016). Bacterial resistance is attributed to genetic modifications, such as gene mutation and lateral gene transfer, that improve bacterial chances of survival in high concentrations of different antimicrobial compounds (Zaman et al., 2017). In addition, persistent infections also cause high levels of morbidity and mortality globally and are an important cause of recurrent infective diseases. Even though persister cells do not depend on genetic modifications, molecular mechanisms are known to link persister cells and resistance, which could promote the spread of antibiotic resistance following persister cell recovery (Fisher et al., 2017).

**FIGURE 2:**
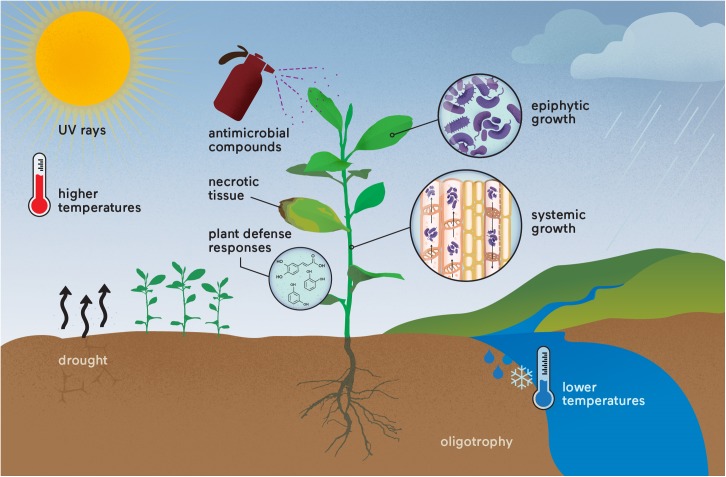
“Super-phytopathogenic” bacteria occurrence in the field. Different stress conditions are already known to affect phytopathogens that could induce resistance and/or persister cell formation. The recurrence of disease outbreaks may result from these genetic and physiological responses, which are still underestimated in both research and crop management. Parallels could be made with the human superbugs.

The existence of resistance and persister cells in phytopathogens might be the reason why eradication of plant diseases has not been successfully attained, despite the massive stress conditions faced by the phytopathogens in the environment, such as UV radiation, temperature, drought, plant defense responses and antimicrobial compounds (Poplawsky et al., 2000; Lin et al., 2001; Barron and Forsythe, 2007; Gunasekera and Paul, 2007; Vriezen et al., 2012; Zhang et al., 2015; Tondo et al., 2016; Leonard et al., 2017) (**Figure 2**). In addition, this might also be an important reason for crop disease outbreaks and recurrence, even with the frequent use of antimicrobial compounds and other management methods in the field (Graham et al., 2004; McGhee and Sundin, 2011; Aćimović et al., 2015; Hippler et al., 2018). One good example of recurrence is in *X. citri*; copper is widely used to control this bacterium, and persister cells are formed under copper stress, allowing regrowth (del Campo et al., 2009). Moreover, resistance to copper is also observed for *X. citri* in the field (Richard et al., 2017a,b; Gochez et al., 2018), leading to a continuously increasing use of copper in the field to control citrus canker. However, similar to human diseases, this infection is recurrent (Graham et al., 2004). This example can also be applied to other plant bacterial diseases where copper or antibiotics do not kill the entire bacterial population and infection is recurrent (Aćimović et al., 2015; Areas et al., 2017; Gochez et al., 2018). Consequently, it is reasonable to say that there is an appropriate condition for the appearance of “super-phytopathogenic” bacteria similar to the human superbugs. However, in contrast to human pathogenic bacteria, where there are many studies focusing on persister cells, the occurrence of persistence in phytopathogens and studies focusing on this phenomenon in the field are sparse. It is imperative to improve knowledge aiming to understand the biological, environmental, and chemical factors that lead to the formation of persister cells in the field and their regrowth. These studies are necessary to develop new sustainable strategies to control persistent infections in agriculture.

## Author Contributions

ADS conceived the manuscript. PM and MM wrote the manuscript. ADS and MT helped to write the manuscript.

## Conflict of Interest Statement

The authors declare that the research was conducted in the absence of any commercial or financial relationships that could be construed as a potential conflict of interest.
